# Local and Systemic Effects of *Porphyromonas gingivalis* Infection

**DOI:** 10.3390/microorganisms11020470

**Published:** 2023-02-13

**Authors:** William A. Chen, Yuetan Dou, Hansel M. Fletcher, Danilo S. Boskovic

**Affiliations:** 1 Division of Biochemistry, Department of Basic Sciences, School of Medicine, Loma Linda University, Loma Linda, CA 92350, USA; 2 Division of Microbiology and Molecular Genetics, Department of Basic Sciences, School of Medicine, Loma Linda University, Loma Linda, CA 92350, USA

**Keywords:** dentistry, inflammation, neutrophils, periodontitis, periodontology, platelets, *Porphyromonas gingivalis*

## Abstract

*Porphyromonas gingivalis*, a gram-negative anaerobe, is a leading etiological agent in periodontitis. This infectious pathogen can induce a dysbiotic, proinflammatory state within the oral cavity by disrupting commensal interactions between the host and oral microbiota. It is advantageous for *P. gingivalis* to avoid complete host immunosuppression, as inflammation-induced tissue damage provides essential nutrients necessary for robust bacterial proliferation. In this context, *P. gingivalis* can gain access to the systemic circulation, where it can promote a prothrombotic state. *P. gingivalis* expresses a number of virulence factors, which aid this pathogen toward infection of a variety of host cells, evasion of detection by the host immune system, subversion of the host immune responses, and activation of several humoral and cellular hemostatic factors.

## 1. Overview

*Porphyromonas gingivalis* is a black-pigmented, gram-negative bacterium that primarily colonizes the subgingival tissues in the oral cavity. This asaccharolytic anaerobe can adapt to and survive in the low oxygen tension conditions characteristic of periodontal pockets [1]. However, growth rates under microaerophilic conditions are not significantly altered from those at anaerobic conditions, suggesting that *P. gingivalis* can tolerate microenvironments with low oxygen [2]. Hemin or heme and vitamin K can be used as growth nutrients [3,4]. However, *P. gingivalis* also metabolizes amino acids (AAs) and peptides for energy and as a supply of carbon [5].

Over 700 bacterial species of diverse microbial flora are estimated to inhabit the oral cavity [6]. New culture-independent and culture-dependent molecular techniques have been developed to help characterize microbial communities. Culture-independent approaches include techniques such as next-generation sequencing (NGS) technologies, such as shotgun metagenomics sequencing, allowing researchers to investigate populations of oral bacteria [7]. Deoxyribonucleic acid (DNA) is extracted from the oral microbiome and fragmented prior to sequencing [8]. Then metagenomic analysis helps to highlight the genomic characteristics and potential functions of oral microbiota [9]. Similarly, meta-transcriptomic analyses of ribonucleic acid (RNA) help to assess gene expression in mixed bacterial populations of the oral cavity [10]. Such techniques have been used to investigate interactions of *P. gingivalis* with various other bacterial species and evaluate its effects on the microbial community within the biofilm environment [11,12].

The new culture-dependent techniques employ a variety of media prior to analysis using sensitive mass spectrometric and sequencing techniques, such as matrix-assisted laser desorption ionization–time of flight mass spectrometry (MALDI–TOF MS) and 16S ribosomal RNA (rRNA) sequencing, to identify bacterial species [13,14]. Future studies can involve such culturomic approaches to characterize particular roles of specific bacteria, such as *P. gingivalis* during the colonization of periodontal tissues and the pathogenesis of periodontitis [15].

The subgingival biofilm, within periodontal pockets, comprises over 300 different species [16]. In this context, *P. gingivalis* is considered to be a late colonizer, often co-aggregating at the top layer with initial and secondary colonizers [17,18]. While most oral microbes are seen as commensal, some, including *P. gingivalis*, are recognized as opportunistic or keystone pathogens [19].

## 2. Virulence Factors

The plasma membrane of *P. gingivalis* acts as a dynamic interface between the oral pathogen and its local environment. Successful acquisition of nutrients facilitates growth, while effective tissue colonization ensures bacterial survival. These are highly dependent on virulence factors expressed or released by *P. gingivalis* (Table 1).

## 3. Capsule

Gram-negative bacteria, including *P. gingivalis*, are covered with cell surface macromolecules termed capsular polysaccharides (CPS) [45]. Bacterial capsules are typically carbohydrate homo- or heteropolymers, composed of repeating monosaccharide units covalently bound through glycosidic linkages [46]. Carboxyl or phosphate groups may be attached to the carbon backbone of the monosaccharide residues, contributing negative charges to CPS [47]. The capsule includes mannuronic acid, glucuronic acid, galacturonic acid, galactose, and 2-acetamido-2-deoxy-d-glucose (N-acetylglucosamine) at a relative molar ratio of 1.2:1.8:1.0:1.0:2.0, respectively [48]. Additionally, the CPS in several gram-negative pathogens are terminally linked to reducing phospholipids, which anchor the glycolipids into the outer membrane [49].

## 4. Capsular Functions

Encapsulation contributes to *P. gingivalis* pathogenicity by enabling the evasion of detection by the immune cells and increasing the resistance to phagocytosis [20]. Furthermore, human gingival fibroblasts, when challenged with *P. gingivalis* lacking a capsule, increased interleukin (IL)-1β, IL-6, and IL-8 cytokine production [50]. The presence of a capsule may help to mask immunogenic bacterial agents, such as adhesins or invasins, and prevent the triggering of some immune responses [51]. In addition to this camouflage-like role, it was suggested that encapsulated *P. gingivalis* are also more invasive, although there are some conflicting reports about this capsular effect. The highest degree of bacterial invasion is observed when human coronary artery endothelial cells are incubated with wild-type *P. gingivalis* compared to other *P. gingivalis* strains [21]. However, it was also reported that mutant non-encapsulated *P. gingivalis* strains were more efficient at invading gingival fibroblasts [52]. In this context, the gram-negative *Neisseria meningitides*, associated with septicemia and meningitis, are known to downregulate capsule synthesis and assembly during adhesion to target cells [53]. A similar mechanism may be utilized during the early stages of *P. gingivalis* infection of subgingival tissue, reducing the capsule production to increase adhesion. Consistent with this, in contrast to *P. gingivalis* cultured from healthy sites, *P. gingivalis* cultured from diseased periodontal sites are highly invasive to KB cells (a subline of HeLa cells) [54]. Overall, however, encapsulated *P. gingivalis* strains are associated with increased virulence, implying that capsule expression contributes to bacterial survival and its ability to impact host immune responses.

## 5. Fimbriae

### 5.1. Major Fimbriae

Characteristic of gram-negative bacterial cells, fimbriae are thin protruding appendages attached to the outer membrane. *P. gingivalis* expresses two distinct types of fimbriae that vary in length, classified as major and minor. The long fimbrial polymer is primarily composed of the repeating fimbrillin (Fim) A subunits [55], which are assembled through a head-to-tail oligomerization [56]. Various *P. gingivalis* strains produce different overall FimA monomer sizes with some amino-terminus sequence heterogeneity [57]. The *fimA* gene, encoding the FimA protein, has six recognized gene variants: type I, Ib, II, III, IV, and V [58], and belongs to a cluster of seven *fim* genes: *fimX*, *pgmA*, and *fimABCDE* [59]. FimB regulates the length of FimA-associated fimbriae. A nonsense mutation in *fimB* leads to the production of extended fimbriae in *P. gingivalis* strains ATCC33277 and 381 [60]. FimC, FimD, and FimE represent accessory proteins, forming complexes that facilitate the biosynthesis of long fimbriae [61,62]. Deficiency of any of the accessory proteins reduces the attachment of *P. gingivalis* mutants to extracellular matrix (ECM) protein fibronectin or to type I collagen [62].

### 5.2. Minor Fimbriae

Minor fimbrial antigen 1 (Mfa1) is the main structural protein of *P. gingivalis* short fimbriae [63]. The *mfa1* gene is part of a four-gene cluster operon comprising *mfa1*-*4*, while the *mfa5* gene appears to be independently transcribed [64]. Formation of short fimbriae involves the polymerization of monomeric Mfa1 subunits via the amino- and carboxy-terminus domains [65]. In addition, short fimbriae also comprise Mfa2-5 protein subunits. Mfa2 is a dual-function protein regulating the fimbrial length and its anchoring to the outer membrane. Mfa3 proteins are localized toward the fimbrial tips and work in conjunction with Mfa4 and Mfa5 to properly assemble the short fimbriae [66]. Mfa4 confers a stabilizing effect during the formation of short fimbriae, while Mfa5 possesses a von Willebrand Factor (vWF) type A domain that mediates protein–protein interactions with Mfa1 [67,68]. For pathogenic bacteria, such as *Enterococcus faecalis* and *Streptococcus agalactiae*, the vWF type A domain containing pilus proteins are known to mediate bacterial cell adhesion to host tissue [69,70].

### 5.3. Functions of Fimbriae

*P. gingivalis* fimbriae serve dual functions during the pathogenesis of periodontitis: bacterial adhesion and invasion. The long and short fimbriae support bacterial colonization of gingival tissue and ensure its survival. First, fimbriae enable bacterial adhesion to host cells. Purified fimbriae from *P. gingivalis* cells adhere to human gingival cells in a concentration and time-dependent manner [23]. Impaired adhesion to human gingival fibroblast and epithelial cells is observed in mutant *P. gingivalis* strains deficient in long fimbriae [71]. Similarly, bacterial adherence to human gingival epithelial cells is inhibited in a double knockout strain of *fimA* and *mfa1* genes [72]. Second, *P. gingivalis* invasiveness is dependent on fimbriae. A FimA deficient strain has diminished potential for bacterial invasion of gingival epithelial and fibroblast cells [22,73]. The invasion of epithelial cells can occur through actin-based cytoskeletal rearrangements, mediated by FimA interactions with surface epithelial β1 integrins [24,74].

Alveolar bone destruction is a hallmark feature of *P. gingivalis*-induced periodontitis. Gnotobiotic rats exhibit significant bone loss after infection with fimbriae expressing wild-type *P. gingivalis* [75]. However, such bone loss is mitigated in the rats that are (a) immunized with purified fimbrial proteins before exposure to *P. gingivalis* or (b) infected with a *fimA* knockout strain [75,76]. Consistent with this, alveolar bone loss is not increased in rats infected with a double knockout strain for *fimA* and *mfa1* [72]. Moreover, purified Mfa1 protein increased the release of the cytokines IL-β, IL-6, and TNFα from isolated murine peritoneal macrophages, consistent with the promotion of a proinflammatory environment [25]. Such chronic inflammation then leads to tissue breakdown facilitating the further bacterial invasion of the subgingival regions [26]. In contrast, downregulation of fimbrial expression reduces *P. gingivalis* pathogenicity.

## 6. Gingipains

Gingipains are surface-expressed or secreted cysteine proteases that can cleave a variety of host proteins in plasma, ECM, and in association with immune cells [31,32,77,78,79,80,81]. Their physiologic effects are functional and focused on bacterial survival. Since *P. gingivalis* is asaccharolytic, other types of biomolecules are used for its energy and carbon supply needs. Gingipains cleave host proteins to generate peptides and amino acids, which in turn supply some of these requirements. Proteolysis of host proteins also facilitates bacterial invasion and colonization of subgingival tissues. Furthermore, gingipains can undermine the host immune responses allowing *P. gingivalis* to evade neutrophil-mediated bacterial clearance. Overall, gingipains play a pivotal role in exerting bacterial pathogenicity and catalyzing the degradation of a broad spectrum of host proteins to support the proliferation and invasion of the periodontium.

### Gingipain Structure

*P. gingivalis* expresses two distinct forms of gingipains, arginine- and lysine-specific, that hydrolyze peptide bonds at carboxy termini of arginine or lysine residues, respectively [82]. This cysteine protease family comprises three related enzymes: a high molecular mass arginine gingipain A (RgpA), arginine gingipain B (RgpB), and a lysine gingipain (Kgp). These gingipains are encoded by *rpgA*, *rgpB*, and *kgp* genes, respectively [83]. The gingipain precursors exhibit similar structural features with a signal peptide attached to an amino-terminus propeptide domain, followed by an arginine- or lysine-specific catalytic domain [84]. Hemagglutinin/adhesin domains are linked to the catalytic domain of RgpA and Kgp at their respective carboxy-termini. RgpB lacks such domains. The four identified hemagglutinin/adhesin domains are designated as Hgp15, Hgp17, Hgp27, and Hgp44 [85]. Hgp15 and Hgp44 are implicated with hemagglutination and with hemoglobin binding activity [27,86]. Hgp17 mediates coaggregation between *P. gingivalis* and *Prevotella intermedia*, suggesting a contributing role in periodontal biofilm formation [87]. Gingipain gene products are inactive zymogens that require catalytic activation following post-translational modifications. Prior to export to the outer membrane, processing of the gingipain gene translation products includes cleavage of the attached signal peptide and propeptide domains as well as the noncovalent association of functional domains [88]. *P. gingivalis* gingipains are expressed in three main forms: membrane-bound monomeric proteins, membrane-bound multimeric complexes, and secreted soluble proteins. Purified RgpA, RgpB, and Kgp are synthesized as single-chain glycoprotein enzymes and expressed along the surface of *P. gingivalis* [89,90,91]. RgpA can interact with Kgp through noncovalent bonds to form large multifunctional complexes [92,93]. Outer membrane vesicles with gingipains can also be secreted into the local environment [94].

Some gingipain expression is central in the development of *P. gingivalis* virulence during infection of the oral cavity. These cysteine proteases mediate tissue colonization to establish a niche in the subgingival regions and degrade host proteins to provide essential nutrients for bacterial growth. Furthermore, gingipain-mediated subversion of the host immune responses promotes the proinflammatory environment that contributes to pathogenic persistence and dysbiosis of the oral microbiota.

## 7. Gingipain Functions

### Tissue Colonization

During the initial stages of infection, gingipains assist bacterial adhesion to subgingival tissues. Wild-type *P. gingivalis* adheres to human oral epithelial cells, while strains deficient in arginine gingipain activity have reduced attachment to these cells [41,95]. ECM cell adhesion molecules (CAMs) located on gingival tissue surfaces mediate such cell-cell interactions. Purified gingipain enzymes were found to bind to CAMs such as fibronectin and laminin [96]. Similarly, in vitro binding activity of wild-type *P. gingivalis* was demonstrated toward type I collagen, fibronectin, and laminin [41]. Reduced binding to these ECM proteins was observed for *P. gingivalis* with inactivated *rgpA* gene. Adherence to immobilized collagen is also diminished in the *rgpA* knockout [33].

Gingipain adhesin domains mediate tissue colonization of *P. gingivalis* in the subgingival sulcus. The binding of adhesin proteins to epithelial cells can be detected even if wild-type *P. gingivalis* is treated with a protease inhibitor, implying that protease functions of gingipains are not involved [97]. However, treatment with an anti-adhesin antibody blocks interactions of epithelial cells with native RgpA adhesin proteins or with wild-type *P. gingivalis* cells [98]. Moreover, gingipain adhesin is known to bind to ECM proteins such as collagen, fibronectin, and laminin [31,99,100].

*P. gingivalis* expressed fimbriae are heavily implicated as primary virulence factors initiating bacterial adhesion to host cells. However, gingipain proteolytic activity plays a role in effective fimbrial function. Post-translational processing of precursor fimbrillin proteins is crucial for proper fimbrial assembly along the outer membrane [101]. Decreased expression of cell surface fimbriae is observed following the inactivation of both the *rgpA* and *rgpB* [42]. Additionally, fimbrial binding to gingival fibroblast cells is increased in the presence of purified gingipains [34]. In this context, pretreatment of fibronectin with purified gingipains also increases binding to fimbriae [102]. This effect can be reduced by the addition of competing L-arginine residues or peptides with exposed arginine residues. Together, these observations imply that proteolytic cleavage of ECM proteins by gingipains potentially unmask cryptic receptors, or cryptitopes, with exposed arginine residues that mediate fimbrial attachment [103]. Thus, gingipains cleave fimbrial precursors during the production and maturation of fimbriae, as well as modify target CAMs, to enhance bacterial adhesion with host cells.

## 8. Tissue Invasion

Following the attachment to cell surfaces, gingipains facilitate the invasion of gingival tissues. Transmission electron micrographs from invasion assays illustrate internalized *P. gingivalis* bacterial cells within oral epithelial cells [104]. Similarly, in vitro fluorescence imaging demonstrates *P. gingivalis* localization within gingival epithelial cells [105]. In contrast, bacterial pretreatment with protease inhibitors significantly reduces such invasion of epithelial cells by *P. gingivalis* [106]. Wild-type *P. gingivalis* infiltrates the entirety of a human oral mucosal tissue model, including internalization within gingival epithelial cells, as well as penetration into the basement membrane and lamina propria [107]. The triple (*rgpA*, *rgpB*, *kgp*) gingipain knockout strain, however, is primarily localized to the epithelial surface. Gingival tissue invasion involves cleavage of CAMs and cytoskeletal remodeling. Increased fibronectin and its fragments are observed in gingival crevicular fluid (GCF) from patients with periodontitis but not from healthy subjects [80]. Similarly, fibronectin degradation is observed when purified arginine gingipains are incubated with human gingival fibroblast cells [108]. Moreover, purified RgpA, RgpB, or Kgp gingipains can cleave isolated human serum fibronectin [80].

Purified RgpA and Kgp can digest laminin directly [31]. However, the most prevalent form of collagen in gingival connective tissues, type I, is resistant to degradation by purified gingipains [109,110]. Nevertheless, *P. gingivalis* can cause connective tissue proteolysis by upregulating activation and downregulating inhibition of matrix metalloproteases (MMPs) [111,112,113,114]. MMPs are proteolytic enzymes that degrade ECM proteins in a calcium and zinc-dependent manner [115]. These metalloproteases can be released by fibroblasts from the periodontal ligament and gingival tissue [114,116].

Gingipains can alter the host cell's cytoskeletal responses to facilitate bacterial invasion. *P. gingivalis*-mediated infection of gingival epithelial cells triggers significant depolymerization of actin and disassembly of microtubules in host cells [24]. Such actin degradation is not observed when a triple gingipain (*rgpA*, *rgpB*, *kgp*) knockout is incubated with human gingival epithelial cells [117]. However, actin cleavage occurs when the gingival epithelial cells are challenged with a double (*rgpA*, *rgpB*) knockout, implying that Kgp is the effective enzyme. This is confirmed by dose and time-dependent degradation of isolated actin by purified Kgp [117]. In contrast, the cleavage of isolated actin by purified RgpA or RgpB is not significant.

Epithelial cells in gingival tissues form tight and adherent junctional complexes via specialized protein–protein interactions [118]. These complexes create homotypic cell–cell adhesions that together constitute a physical barrier to pathogens [119]. *P. gingivalis* is known to break down the intercellular junctional complexes, thus disrupting this important epithelial barrier function [120]. Incubation of *P. gingivalis* with Madin-Darby canine kidney (MDCK) cells results in reduced labeling intensity of occludin and E-cadherin [121]. Western blots confirm this effect with concentration-dependent degradation of junctional proteins. In this context, gingipains are the likely enzymes capable of degrading epithelial junctional proteins. All three forms of purified gingipains (RgpA, RgpB, and Kgp) are known to catalyze the hydrolysis of immunoprecipitated E-cadherin in a concentration-dependent manner [122].

Gingipain-mediated degradation of epithelial CAMs, as well as hydrolysis of actin and junctional proteins, provide *P. gingivalis* with access to underlying connective tissue. This creates an environment conducive to bacterial colonization of the periodontium, an important step in the pathogenesis of periodontitis. The compromising of the structural integrity of gingival epithelium may lead to further tissue destruction, a hallmark feature of *P. gingivalis*-induced periodontitis [123]. Immunization of a mouse model with purified RgpA or RgpB, prior to subcutaneous inoculation of *P. gingivalis*, inhibits abscess formation and mortality [124]. This implies that gingipain immunization reduces the pathogenicity of *P. gingivalis* and protects mice against tissue invasion. Similarly, bacterial preincubation with cysteine proteinase inhibitors prevents the development of necrotic lesions along the mouse abdomen and protects the mice from death [125]. Additionally, death is not observed in mice infected with low concentrations of the double gingipain knockout (*rgpA, rgpB)* strain [126]. Even at higher doses, survival rates for mice infected with this double-knockout strain are significantly higher compared to mice infected with the wild-type strain. Similarly, infection with a *kgp* gene knockout strain also improves survivability in mice. Taken together, either (a) gingipain gene inactivation or (b) pretreatment of *P. gingivalis* culture with gingipain inhibitors attenuates bacterial virulence resulting in diminished protein degradation and reduced tissue invasion.

## 9. Nutrient Acquisition

Iron is an essential metal required for a wide range of physiological processes, including DNA replication/repair, mitochondrial function, myelin synthesis, and red blood cell (RBC) mediated oxygen transport [127,128,129,130]. For most organisms, iron bioavailability is a major requirement for cell proliferation and metabolic maintenance [131,132]. Vertebrates produce several high-affinity iron-binding proteins, such as ferritin, transferrin, hemoglobin, and hemopexin, for transport, sequestration, and prevention of cytotoxicity [133]. Free iron is in a redox-active state, able to support the Fenton reaction and produce reactive oxygen species (ROS), including free radicals [134]. Heme is composed of a porphyrin ring, comprising four pyrrole rings linked by methene bridges, with a bound iron at the center of the structure [135]. *P. gingivalis* is a porphyrin auxotroph lacking the enzymatic machinery for porphyrin biosynthesis [5]. As a result, this oral pathogen depends on heme uptake from exogenous sources to meet its needs for iron and for porphyrin-linked iron, both of which are vital nutrients for bacterial growth and proliferation [136].

RBCs represent a major iron reserve in the host, which can serve as an accessible iron source for the nutritional requirements of *P. gingivalis*. Gingipains play a central role in iron acquisition, starting with gingipain-mediated hemagglutination and hemolysis. RgpA and Kgp contain four hemagglutinin/adhesin domains, which mediate *P. gingivalis* interactions with RBCs [27]. Purified RgpA or Kgp is capable of inducing agglutination of RBCs, while RgpB is not [35]. The triple gingipain (*rgpA*, *rgpB*, *kgp*) knockout strain is typically characterized by inhibited RBCs hemagglutination potential, while single RgpA or Kgp knockout strains support reduced RBC binding [137]. Agglutination of RBCs is followed by the formation of small aggregates, allowing enzymes to slowly lyse bound RBCs, releasing their hemoglobin. In the presence of protease inhibitors, the hemolysin activity of wild-type *P. gingivalis* is suppressed [138]. Furthermore, Kgp-deficient strains are characterized by their significantly reduced hemolytic function [139]. However, degradation of RBCs is not inhibited when the triple gingipain (*rgpA*, *rgpB*, *kgp*) knockout is used, suggesting that gingipains may not be the primary sources of the hemolytic function [137].

Hemoglobin is a globular heterotetrameric protein comprising two α and two β polypeptide chains, each of which noncovalently binds to its own single heme prosthetic group [140]. Gingipains can interact with and degrade the hemoglobin protein, releasing the heme, including its coordinated iron. High binding affinity for hemoglobin was reported for both RgpA and Kgp, and a somewhat weaker interaction was observed for RgpB [43]. Furthermore, purified hemagglutinin/adhesin domains from both RgpA and Kgp have a high binding affinity for hemoglobin [100]. This suggests that the RgpA or Kgp hemagglutinin/adhesin domains mediate protein-protein interactions between gingipains and hemoglobin. Further, the Hgp15, or hemagglutinin/adhesin domain 2, a conserved domain expressed in both RgpA and Kgp, can interact with the heme group [141]. However, unlike purified RgpA or RgpB, only purified Kgp completely degrades hemoglobin in a time-dependent manner [142]. Nevertheless, RgpA and Kgp appear to form large multimeric protease complexes, which are effective for heme acquisition [143]. Gingipain-associated hemagglutinin/adhesin domains facilitate the adhesion of the complex to hemoglobin, localizing target protein within the range of Kgp for rapid degradation. While gingipains are able to degrade other iron-binding proteins, including transferrin and hemopexin, hemoglobin remains the preferred source of iron for *P. gingivalis* [77,142,144].

As an asaccharolytic pathogen, *P. gingivalis* relies on host proteins to supply its metabolic energy and nutrient needs. Peptide fragments appear to be the preferred nutrient for bacterial growth. Minimal metabolism of free AAs is observed in *P. gingivalis* culture media, with none becoming fully depleted [145]. This limited utilization of free AAs may be due to a lack of suitable transport systems [146]. Washed *P. gingivalis* bacterial cells incubated with peptides, and free AA, preferentially hydrolyze peptides containing aspartate, glutamate, leucine, and valine [28]. In contrast, free AAs tend to be minimally used [147]. Gingipains can facilitate the breakdown of plasma proteins, such as serum albumin or transferrin, into peptides for use as carbon and nitrogen sources as well as for metabolic energy. The presence of a protease inhibitor in the *P. gingivalis* culture medium, in addition to human serum albumin, inhibits bacterial growth and partially reduces the ability to degrade the protein [78]. Furthermore, the single gene (*rgpA* or *kgp*), double gene (*rgpA*, *rgpB*), or triple gene (*rgpA*, *rgpB*, *kgp*) knockout strains are unable to grow in human serum supplemented with hemin [137]. However, bacterial growth is restored for all mutant strains after the addition of exogenous peptides. Nevertheless, doubling times are prolonged for the gingipain knockout strains, consistent with a contributing role of gingipains in the acquisition of nutrients for optimal proliferation [137].

## 10. Altered Host Immune Responses to *P. gingivalis*

Neutralizing host immune defenses is necessary for *P. gingivalis* to successfully colonize the periodontium and proliferate within the subgingival region. To this end, the bacteria (a) evade the host immune response and (b) subvert the immune cell-mediated bacterial clearance. Avoiding detection by the host immune system is pivotal to bacterial survival within the oral microenvironment. However, *P. gingivalis* can also manipulate the host's inflammatory response. Initial infection in the oral cavity induces the production of a multitude of chemokines and cytokines. Increased IL-1β, IL-6, IL-8, and tumor necrosis factor (TNF)-α are released following incubation of wild-type *P. gingivalis* with cultured human gingival fibroblast cells, oral epithelial cells, or periodontal ligament cells from healthy subjects [148,149,150]. Mice infected with wild-type *P. gingivalis* have significantly higher serum levels of IL-1β, IL-6, IL-8, and TNF-α, compared to controls or compared to mice infected with a mutant strain deficient in functional fimbriae [151]. Elevated IL-1β, IL-6, IL-8, IL-17, and TNF-α are measured in GCF, inflamed gingival tissues, or in serum obtained from patients with periodontitis [152,153,154,155]. In this context, tissue cytokine concentrations are dependent on the biopsy distance from the infection site, suggesting that cytokine expression is influenced by the progression and severity of periodontitis [154].

## 11. Disruption of Host Inflammatory Responses to *P. gingivalis*

Inflammation is a physiologic host response to pathogenic infection. Infected cells secrete proinflammatory mediators that serve as chemotactic signals to recruit immune effector cells, which mediate bacterial clearance. IL-1β is a proinflammatory cytokine commonly secreted by monocytes or macrophages [156]. It is also produced by other cell types, including gingival epithelial and fibroblast cells [157,158]. In response to pathogenic stimuli, this primary cytokine regulates the initial stages of inflammation by inducing IL-6 and IL-8 production via TLR and nuclear factor-κβ (NF-κB) signaling pathways [159,160].

IL-6 is a proinflammatory cytokine secreted by gingival and periodontal ligament fibroblasts [161,162]. *P. gingivalis*-induced IL-6 expression increases bone resorption by downregulating osteoprotegerin (OPG) [158]. OPG is released by osteoblast cells as a decoy receptor that binds to and inactivates receptor activator of nuclear factor κβ ligand (RANKL) function [163]. RANKL binding to the receptor activator of nuclear factor κβ (RANK) receptor triggers a signaling cascade that activates mitogen-activated protein kinase (MAPK) and other transcription factors involved with osteoclast proliferation [164]. Gingipain-mediated disruption of the RANKL/OPG ratio upregulates RANKL function and drives the system to favor osteoclast differentiation, a predominant cell-type responsible for bone matrix degradation [165,166]. The resulting alveolar bone loss is commonly associated with periodontitis [167].

IL-8 is a chemokine secreted by gingival epithelial cells, gingival fibroblasts, and periodontal ligament fibroblasts [168,169]. It is a dual-function inflammatory mediator involved in neutrophil chemotaxis and activation. IL-8 secretion forms a chemoattractant gradient that functions as a host signaling mechanism to recruit neutrophils to the infection site [170]. Additionally, IL-8 triggers G protein-coupled receptor (GPCR)-phospholipase C (PLC) signaling pathways to activate neutrophils [171]. Mobilization of intracellular calcium stores contributes to neutrophil shape polarization, adhesion, degranulation, or oxidative burst [172,173,174].

TNF-α is a pleiotropic proinflammatory cytokine primarily secreted by activated macrophages [175]. It works synergistically with RANKL to upregulate RANK receptor expression [176]. TNF-α and RANKL signaling pathways trigger NF-κB activation to induce transcription of other transcription factors (nuclear factor of activated T-cells, cytoplasmic 1 (NFATc1) and c-Fos) and protease genes (cathepsin K and MMP-9), involved in osteoclastogenesis and bone resorption, respectively [177]. Expression of NFATc1 and c-Fos messenger RNA (mRNA) is increased in skull bones from mice injected with *P. gingivalis* lipopolysaccharide (LPS) [178]. In the presence of TNF-α, human periodontal ligament cells have increased expression of MMPs [179]. Furthermore, bone resorption is significantly reduced in *P. gingivalis*-infected cathepsin K knockout mice [180].

As *P. gingivalis* infection progresses deeper into the periodontium, the release of chemokines and cytokines may be altered. Membrane-expressed gingipains can exploit the host inflammatory responses by degrading proinflammatory cytokines. Human gingival epithelial cells treated with wild-type *P. gingivalis* elicit the release of IL-1β, IL-6, and IL-8 [30]. However, IL-6 and IL-8 are rapidly digested within an hour, while IL-1β degradation occurs over time. Treatment of gingival epithelial cells with the triple gingipain (*rgpA*, *rgpB*, *kgp*) knockout strain, but not with the double (*rgpA*, *rgpB*) knockout, abolishes cytokine degradation. Similarly, transient increases in IL-1β, IL-6, or IL-8 levels are observed upon incubation of *P. gingivalis* with human gingival fibroblasts [181]. However, treatments with heat-killed *P. gingivalis* or with gingipain-deficient strains lead to significantly higher IL-6 and IL-8 secretion from fibroblasts.

Additional inflammatory gingipain targets, including chemokine (C-C motif) ligand (CCL) 2, CCL5, chemokine (C-X-C motif) ligand (CXCL) 1, and CXCL10, are suppressed following a challenge of human gingival fibroblasts with *P. gingivalis* [182]. In contrast, chemokine secretion is observed in fibroblast cell cultures after a challenge with heat-killed *P. gingivalis*. Kgp is implicated as the primary gingipain involved in disrupting the cytokine signaling network since minimal effects are observed when epithelial cells are treated with a Kgp-null strain [30]. Taken together, expressed *P. gingivalis* gingipains promote a persistent proinflammatory environment within the subgingival region to extract nutrients from host proteins and evade host immune cells.

## 12. Subversion of Host Immune Responses by *P. gingivalis*

Successful colonization of the periodontium is crucial to establishing a subgingival niche and fostering bacterial proliferation. Subsequently, long-term bacterial survival requires evasion of the host immune responses to avoid bacterial killing. Impaired immune responses enable a proinflammatory environment that favors persistent infection, characteristic of chronic periodontitis.

The complement system, integral to the host immune defenses, comprises a group of plasma proteins that generally contribute to pathogen identification and elimination. However, wild-type *P. gingivalis* viability in human serum is not significantly affected by incubation with functionally competent complement factors [137]. Single (*rgpA, rgpB,* or *kgp*) or double (*rgpA, rgpB*) gingipain gene inactivation significantly reduces *P. gingivalis* survival in human serum, suggesting a contributing role for these cysteine proteases in disruption of the complement bactericidal functions. Gingipains cleave and degrade complement components C3, C4, and C5 [183]. Purified gingipains cleave isolated C3 into C3a- and C3b-like peptides. C3a is then susceptible to further time-dependent degradation into nonfunctional fragments. In addition, purified C3 is not efficiently cleaved when incubated with a RgpA deficient *P. gingivalis* strain [36]. Furthermore, this mutant strain is associated with significantly higher levels of bound C3 protein, and the addition of a protease inhibitor increases C3 binding to wild-type *P. gingivalis*. Uptake of the RgpA deficient strain by polymorphonuclear leukocytes (PMNs) is also significantly improved in the presence of complement factors [36]. However, PMN-mediated uptake of the wild-type strain is observed only at higher complement levels, consistent with the anticipated degradation of complement factors.

C3a is a proinflammatory anaphylatoxin mediating the degranulation of eosinophils and chemotaxis of mast cells [184,185]. The α-chains of C3, C4, and C5 can be proteolytically activated by low concentrations of purified RgpA, RgpB, and Kgp [32]. However, at higher levels, all three gingipains further cleave C3, C4, and C5 into nonfunctional fragments. Pretreatment of human serum with wild-type *P. gingivalis* improves the survival of subsequently added *Escherichia coli* [32]. Similarly, pretreatment of human serum with purified RgpA, RgpB, or Kgp increases the *E. coli* viability in a concentration-dependent manner. The reduction of the bactericidal effect of human serum is most and least efficiently achieved by RgpA and Kgp, respectively [32].

C4b-binding protein (C4BP) is a circulating inhibitor of the complement system [186]. C4BP inhibits the assembly of C3 convertase while also serving as a cofactor for proteolytic inactivation of C3b and C4b [186,187]. The C1 complex, a multimeric protease complex comprising one C1q molecule as well as two C1r and two C1s molecules, cleaves C2 and C4 into C2a and C2b as well as C4a and C4b, respectively [188]. The C2a and C4b fragments interact to form the C3 convertase complex (C4b2a) of the classical pathway [188]. In turn, the C3 convertase cleaves C3 to produce C3a and C3b fragments. Of these, the C3b serves as an opsonizing agent targeting pathogens for phagocytosis [189]. Furthermore, C3b also mediates the cleavage of C5 into C5a and C5b, allowing C5b to interact with C6-9 to form a membrane attack complex [188]. This complex creates a pore through the targeted cell membrane resulting in bacterial lysis. Surface-bound C3b on pathogens can also interact with factor B, a serine protease, to create an alternate C3 convertase complex (C3bBb) from the alternative pathway [190]. This C3bBb complex can cleave C3 to amplify the initial complement activation signal.

C4BP can interact with the hemagglutinin/adhesin domains of RgpA gingipain [191]. In addition, a two-fold increase in surface deposition of C9 on *P. gingavlis* occurs following bacterial incubation with human serum deficient in C4BP. Conversely, exogenously added C4BP reduces C9 surface accumulation on *P. gingivalis* to levels seen with normal human serum. The binding of *Haemophilus influenzae* to C4BP does not affect the cofactor function of the complement system inhibitor [192]. Rather, in the presence of C4BP, reduced C3b accumulation is observed on the pathogen surface, consistent with ongoing degradation of C3b and C4b [192]. Similarly, RgpA-mediated binding to C4BP may provide *P. gingivalis* with an evasion mechanism that attenuates host immune defenses by downregulating C3b-mediated opsonization and phagocytosis.

## 13. Gingipains Attenuate Neutrophil Function

The release of chemokines and cytokines at an infection site leads to the recruitment of neutrophils, which serve as the host’s first line of defense against pathogenic bacteria [193]. *P. gingivalis* can impair bacterial clearance by degrading proinflammatory mediators involved with neutrophil recruitment. Reduced levels of secreted IL-8 are observed in human gingival epithelial cells following incubation with *P. gingivalis* [194]. In addition, when oral epithelial cells are challenged with *P. gingivalis*, the expected IL-8-mediated neutrophil transmigration through the epithelial layer becomes attenuated [195].

Surface-expressed gingipains can mediate *P. gingivalis* virulence by disrupting neutrophil recruitment and neutrophil functions at the infection site. The release of TNF-α and several interleukins contributes toward neutrophil recruitment. PMN responses are reduced in mice deficient in TNF receptors following wild-type *P. gingivalis* infection [196]. Moreover, TNF-α digestion is observed to be dose and time-dependent when murine fibroblasts are treated with purified RgpA or RgpB [197]. Similarly, purified RgpA or Kgp degrades recombinant IL-8(77), a mature 77 AA cytokine isoform, in a time-dependent manner [198].

Oral inoculation of *P. gingivalis* in mice elicits a proinflammatory response with the release of several cytokines, including IL-17 [199]. However, IL-17, which is involved with neutrophil recruitment, is susceptible to Kgp-mediated cleavage in a concentration and time-dependent manner [200,201]. Furthermore, neutrophil accumulation in the maxillary gingival tissue is comparable in the IL-17 receptor-deficient mice, whether infected with wild-type *P. gingivalis* or sham infected [202]. Similarly, after another infection, the gingival tissues from IL-17 receptor knockout mice have reduced neutrophil infiltration, comparable to sham-infected IL-17 receptor-deficient mice. However, following a third inoculation, wild-type mice infected with wild-type *P. gingivalis* have significant neutrophil infiltration into the gingival epithelium and adjacent connective tissues compared to sham-infected mice [202]. This implies that *P. gingivalis* tends to suppress the IL-17-mediated signaling and the subsequent neutrophil infiltration.

Taken together, gingipains help *P. gingivalis* to exploit host inflammatory and neutrophil responses. The bacterial infection triggers neutrophil recruitment via chemokine/cytokine release. *P. gingivalis* expressed gingipains degrade inflammatory mediators to attenuate neutrophil functions and promote a chronic proinflammatory environment. In turn, the inflammation-mediated breakdown of gingival tissue provides essential nutrients for bacterial growth.

The glycosylated transmembrane glycoprotein CD99 is expressed on the surface of gingival epithelial, gingival fibroblast, and endothelial cells [203,204]. CD99 has a role in neutrophil transmigration between endothelial cells [205]. Gingipains impair neutrophil adhesion to host cells by interacting with and cleaving CD99. Treatment of human umbilical vein endothelial cells (HUVECs) with an agonistic anti-CD99 monoclonal antibody (mAb) leads to increased expression of CAMs, including endothelial leukocyte adhesion molecule-1 (ELAM-1), vascular cell adhesion molecule-1 (VCAM-1), and intercellular adhesion molecule-1 (ICAM-1) [204]. In contrast, treatment of HUVECs with purified RgpA or Kgp digests CD99 and reduces CAM expression in a dose and time-dependent manner. RgpA appears to be the more efficient protease in this context. PMN binding to HUVECs is reduced in the presence of purified RgpA [204]. However, PMN adhesion is improved following HUVEC treatment with RgpA and an agonist anti-CD99 mAb or following treatment with both a proteinase inhibitor treated RgpA and the mAb agonist. Thus, the addition of gingipains preincubated with a proteinase inhibitor and treatment of HUVECs with the agonist anti-CD99 mAb reduces CD99 proteolysis and increases CAM expression [204]. Together, these observations imply that gingipain-mediated CD99 hydrolysis, along with reduced CAM expression, disrupts the recruitment of leukocytes.

Complement C5 is proteolytically activated to form C5a and C5b fragments. Gingipains are able to cleave C5, releasing C5a, which can serve as an anaphylatoxin and contribute to neutrophil chemotaxis [183]. This proinflammatory mediator establishes a chemoattractant gradient to direct migrating neutrophils toward sites of bacterial infection [206,207]. At low gingipain concentrations, purified C5a tends to be resistant to further degradation by purified RgpA, RgpB, or Kgp [32]. Increasing gingipain concentration, however, promotes further digestion of purified C5a until nonfunctional fragments are produced.

*P. gingivalis* subverts neutrophil antimicrobial functions by manipulating the complement system and the TLR signaling pathway. Cleavage of C5 by *P. gingivalis* gingipains produces endogenous C5a, which interacts with the C5a receptor (C5aR), to inhibit toll-like receptor (TLR) 2 mediated induction of IL-12 [208]. IL-12 is a cytokine implicated in the recruitment of neutrophils for phagocytic bacterial clearance [209]. However, if wild-type mice are injected with a gingipain deficient *P. gingivalis* strain, exogenously added C5a promotes bacterial survival by reducing neutrophil-mediated phagocytosis [199]. Conversely, bacterial viability is significantly reduced if *P. gingivalis*-infected wild-type mice are treated with a C5aR antagonist or an anti-TLR2 mAb [199]. In addition, lower bacterial cell counts are measured in C5aR or TLR2 knockout mice infected with *P. gingivalis*. Similarly, bacterial killing is increased when isolated neutrophils are challenged with *P. gingivalis* in the presence of a C5aR antagonist or with an anti-TLR2 mAb [199]. Furthermore, the total viable oral bacterial counts tend to be reduced in C5aR knockout mice infected with wild-type *P. gingivalis* [210]. Clearly, the communication between C5aR and TLR2 receptors plays a protective role in *P. gingivalis* survival.

Gingipain-mediated C5aR cleavage can be observed when isolated human neutrophils are incubated with purified Kgp [44]. Prior pretreatment of Kgp with proteinase inhibitors suppresses the C5aR degradation. Such C5aR digestion diminishes calcium flux and MPO release, implying Kgp-mediated functional inactivation of neutrophils. Together, gingipain-mediated C5aR degradation represents an alternative form of *P. gingivalis* virulence, attenuating neutrophil function and enhancing bacterial survival. Suppression of the neutrophil antimicrobial function may further lead to the overt growth of other common bacterial species in oral biofilm. Consequently, this can remodel the host commensal microbiota into a dysbiotic state favoring proinflammatory conditions and further aggravating periodontitis's pathogenesis.

Myeloid differentiation primary response 88 (MyD88) is a downstream adaptor protein that mediates TLR2 signaling for the induction of proinflammatory cytokines and for the host inflammatory response [211]. This suggests that MyD88 may not contribute to *P. gingivalis* evasion of neutrophils. On the contrary, *P. gingivalis* viability is higher in MyD88 knockout mice [199]. In addition, the bacterial killing of *P. gingivalis* via phagocytosis is MyD88 dependent [212]. However, time-dependent degradation of the MyD88 protein is observed when isolated neutrophils are incubated with *P. gingivalis* [190]. Moreover, this *P. gingivalis*-mediated digestion of MyD88 is reduced after treatment with a C5aR antagonist or with an anti-TLR2 mAb [199]. This implies that *P. gingivalis* survival involves the exploitation of the C5aR-TLR2 receptor crosstalk and inactivation of the MyD88 protein.

## 14. *P. gingivalis* Induced Periodontitis

Periodontitis is a proinflammatory state mediated by a pathogenic infection within subgingival tissues. The pathophysiology of periodontitis includes a chronic inflammatory environment that may ultimately progress to a breakdown of gingival tissues, including periodontal ligament and destruction of supporting structures for teeth [213]. Gradual loss of gingival epithelial attachment to the tooth enamel surface creates deep periodontal pockets that enable further accumulation of biofilm [214].

Removal of the oral biofilm is key to reducing the tissue destruction associated with periodontitis. Scaling and root planing (SRP) remains the gold standard for non-surgical therapy in patients [215]. However, bacterial re-colonization continues to be a limitation of SRP treatment [216]. Other therapeutic approaches are being investigated, including the use of biotics (prebiotics, probiotics, paraprobiotics, lysates, and post-biotics) and various natural compounds [217,218]. Further investigations into such adjunct therapies may provide additional options for controlling microbial biofilm characteristics and modifying clinical outcomes of oral infections, including those with *P. gingivalis*.

Contributing factors to the formation of oral biofilm include increased opportunistic bacterial colonization, periodontitis, dental prosthetics, poor oral hygiene, and smoking [219,220,221]. Specifically, implants may be associated with increased biofilm formation, inflammation, and periodontitis [222]. In such circumstances, supportive periodontal therapy and preventative oral hygiene practice can enhance the success rate of dental prostheses [223,224,225]. Additionally, in order to control microbial growth, new approaches are being explored, including a variety of nanotechnologies [226,227,228].

Biofilm formation comprises cell-to-cell interactions among multiple bacterial species. Early colonizers of the tooth surface include gram-positive anaerobic bacteria such as *Actinomyces* (*A. oris*), *Streptococcus* (*S. gordonii* and *S. mutans*), or *Veillonella* (*V. denticariosi* and *V. parvula*) [229,230]. The abundance of *Actinomyces* sp. and *Streptococcus* sp., based on meta-transcriptome analyses of human supragingival dental biofilm, was 3–12% and 12–19%, respectively [231]. Following their attachment to the pellicle-coated surface of teeth, initial colonizers can facilitate interactions with late colonizers, such as *P. gingivalis*. The surface-expressed polypeptides of *S. gordonii*, Streptococcal surface protein A (SspA), and SspB interact with Mfa1, a protein component of the *P. gingivalis* fimbriae [232,233]. Mfa1 binds to the SspB adherence region (BAR), a discrete region on SspB [234].

Secondary colonizers, such as *Prevotella intermedia*, *Aggregatibacter actinomycetemcomitans*, and *Fusobacterium nucleatum*, can interact with early colonizers [235]. Subsequently, late colonizers can bind to these secondary colonizers. High interactions between *F. nucleatum* and *P. gingivalis* are observed in coaggregation assays [236]. *P. gingivalis* demonstrated reduced integration into biofilms formed by mutant *F. nucleatum* strains deficient in the outer membrane proteins, fibroblast activation protein 2 (Fap2), and arginine (R)-inhibitable adhesin (RadD) [236].

Interactions between *P. gingivalis* and *F. nucleatum* involve a galactoside moiety and a lectin-like adhesin (FomA), respectively [237,238,239]. Furthermore, the CPS and LPS isolated from *P. gingivalis PK 1924* (serotype K5) can bind to *F. nucleatum* [240]. Consequently, the role of these ‘bridge’ bacteria is to mediate the coaggregation of early and late colonizers [241].

Together, dental biofilm encompasses a diverse bacterial community of over 300 species [16]. Keystone pathogens, such as *P. gingivalis*, transform the biofilm microbiota into a dysbiotic community, which undermines the host immune response and exploits the inflammatory responses to infection. Biofilm buildup propagates persistent chemokine and cytokine production, which is associated with bacterially induced-inflammation of gingival tissues [242]. Ultimately, the diseased pathological state of the periodontium is characterized by irreversible tissue destruction and alveolar bone loss.

## 15. Pathogen Mediated Dysbiosis

During biofilm formation, *P. gingivalis*, as a late colonizer that adheres to earlier colonizers, is identified as a keystone pathogen implicated in the pathogenesis and progression of periodontitis [18,243]. The polymicrobial dysbiosis model implies that a synergistic equilibrium exists between host gingival tissue and the microbial community [244]. Under physiologic conditions, the oral microbiota comprises heterotypic microbes residing in a controlled symbiotic environment. Host inflammatory and immune responses regulate excessive bacterial proliferation and neutralize overt bacterial pathogenicity [245]. Ordinarily, such homeostasis between the host and the commensal microbiota helps to maintain a balanced state of periodontal health [246]. However, during periodontitis, infectious microbes, such as *P. gingivalis*, disrupt this homeostatic balance and shift the commensal microbial community to a pathogenic state [243]. Even at low abundance, *P. gingivalis* mediates a reinforcing cycle of periodontal dysbiosis leading to enhanced bacterial pathogenicity [247]. This opportunistic pathogen manipulates host responses, locally attenuating the immune system while avoiding total immunosuppression [248]. Chronic infection promotes a continuing proinflammatory environment, including a prominent role of gingipains in the enhanced destructiveness of *P. gingivalis*. Inflammation and gingipain-mediated degradation of gingival tissue proteins provide peptides, iron, and other nutrients crucial for bacterial growth and further progression of infection.

Dysbiotic polymicrobial communities characteristically develop increased reliance on the nutrients from the serum-like transudate produced during periodontal inflammation [249]. The adoption of a proteolytic phenotype enables all members of this community to thrive. This is in clear contrast to growth limitations when individual members are tested in isolation. Gingipain expression from *P. gingivalis* contributes to the growth of such a microbial community. However, the expression and release of such proteases in the periodontal pocket also appear to be coordinated via signaling from other community members [249]. This microbially driven feedforward inflammatory loop implies a symbiotic enhancement of the overall virulence potential for progressively faster tissue breakdown and microbial growth [250]. Moreover, interactions of *P. gingivalis* with the host as well as other microbial surfaces, are further aided by the adhesive properties of fimbriae [251]. Consequently, the microbial biofilm becomes difficult to displace, leading to continued invasion and persistent tissue destruction.

In turn, the host tissues respond by upregulating the expression of various genes, including ferric ion binding protein, several proto-oncogenes, an ankryn repeat, and a β-enolase [252]. The host iron-binding protein may compete with the microbial community for its iron requirements. The overexpression of the ankryn repeat is commonly associated with various diseases, such as cancer or cardiovascular disorders [253,254]. Similarly, β-enolase has been associated with metabolism in cancer cells [255]. Together, these trends suggest that chronic inflammation may be associated with increased risks for other diseases, possibly including cancer.

## 16. *P. gingivalis* and Coagulation

The pathogenicity of *P. gingivalis* can potentially extend beyond the oral cavity. Tissue damage from routine oral hygiene practices or dental procedures may facilitate the entry of the periodontopathogen into the systemic circulation [256]. *P. gingivalis* can trigger the activation of prothrombotic mediators, including platelets, increasing the risk for thrombosis [120]. Concentration-dependent shortening of plasma clotting time is observed when human plasma is incubated with purified RgpA or RgpB [37]. *P. gingivalis* expressed gingipains are cysteine proteases, which can activate plasma serine protease coagulation factors [120]. Purified RgpA proteolytically activates factor IX (FIX), factor X (FX), or prothrombin in a concentration and time-dependent manner [37,38,39]. In contrast, purified RgpB cleavage of inactive zymogens yields minimal to no activated factor IX (FIXa), activated factor X (FXa), or thrombin [37,38,39]. The addition of phospholipids and calcium ions, two contributing clotting cofactors, further enhances the RgpA-mediated activation. Moreover, in the presence of phospholipids and calcium ions, RgpA catalytic efficiency (k_cat_/K_m_) of FIX activation is comparable to that observed for a physiological activator, activated factor VII (FVIIa)-tissue factor (TF) complex [39]. However, RgpA is less efficient at FX or prothrombin activation compared to FVIIa-TF or FXa-activated factor V (FVa) complex, respectively [37,38]. Several snake venoms contain enzymes known to activate coagulation factors, including FX or prothrombin [257,258]. In this context, RgpA-mediated FX activation is comparable to that of Russell’s viper venom [37]. In addition, the prothrombin activation rate by RgpA is higher compared to *Notechis scutulus scutulus* venom but lower compared to venom from *Oxyuranus scutellatus* [38]. Taken together, FIX, FX, and prothrombin activation by RgpA may be contributing factors in thrombin production.

## 17. The Role of Gingipains in Platelet Function

Bacterial infection is often transient for individuals with a robust immune system. However, serious thrombotic complications can develop from persistent infection, including infective endocarditis and sepsis-associated disseminated intravascular coagulation [259]. A distinct feature is the bacterially mediated platelet activation leading to the formation of intravascular thrombi [260]. *P. gingivalis* interaction with platelets can induce platelet activation and subsequent aggregation [261]. Intracellular calcium mobilization is associated with platelet activation [262]. Consistent with this, if treated with live *P. gingivalis*, isolated platelets undergo intracellular calcium mobilization [261]. However, this does not occur if resting platelets are treated with heat-killed bacteria or with the double (*rgpA* and *rgpB*) gingipain knockout mutant. A single (*kgp*) gingipain mutant did elicit changes in intracellular calcium levels, however, this was significantly lower compared to platelet exposure to the wild-type strain. Similarly, platelet aggregation is observed following the incubation of *P. gingivalis* with isolated platelets. However, platelet aggregation depends on the ratio of platelet/bacteria, consistent with the possibility of either a threshold phenomenon or multiple competing platelet interactions. In whole blood, platelet expression of CD62P, an adhesion molecule expressed on surfaces of activated platelets, increases following preincubation of high *P. gingivalis* colony-forming units (CFU) with or without subsequent ADP stimulation [263]. Conversely, there is a trend towards higher CD62P expression even in response to low *P. gingivalis* CFU, particularly as preincubation time is extended. This suggests a dose and a time dependence for the impact of *P. gingivalis* preincubation on platelet surface CD62P expression.

High levels of *P. gingivalis* may promote an excitable state in platelets that results in rapid activation following subsequent interaction with physiologic agonists. At lower *P. gingivalis* levels, platelet responses may be triggered with prolonged preincubation times. In this context, whole blood from generalized aggressive periodontitis and periodontitis patients is associated with higher platelet activation [264,265]. Moreover, robust platelet aggregation is observed after incubation of *P. gingivalis* with whole blood from patients with the peripheral arterial disease (PAD) [266]. Similarly, agonist-dependent increases in platelet P-selectin expression are observed after systemic *P. gingivalis* infusion into rats [267]. Preincubation of *P. gingivalis* with whole blood also impacts platelet plug formation under shear conditions [268]. Extending *P. gingivalis* preincubation times past 7.5 min significantly reduces the time for platelet plug-mediated aperture occlusion in the Platelet Function Analyzer (PFA-100). Thus, platelet plug formation time in whole blood is affected both by the *P. gingivalis* concentration and by the duration of bacterial preincubation.

Interestingly, a prolongation of the occlusion time can be observed at certain *P. gingivalis* levels below those needed for the occlusion time shortening [268]. This is explainable either (a) by ineffective platelet activation or (b) by alternate platelet activation pathways. During the bacterial preincubation phase, platelets may become activated in response to interaction with *P. gingivalis*. However, the activated platelets may be insufficient to trigger full platelet aggregation. Consequently, the spent activated platelets become refractory to platelet plug formation, leading to a prolonged occlusion time. Alternatively, if *P. gingivalis* is capable of interacting with multiple platelet activation pathways with characteristic interaction affinities, then multiple platelet functions could be triggered in a concentration-dependent manner. As a result, *P. gingivalis* in whole blood may trigger a variety of time-dependent processes, some of which are possibly functionally opposing [268].

Platelets are involved in a variety of ways with leukocyte functions, including those of neutrophils. Platelet-neutrophil interactions are believed to be mediated by an interaction between platelet P-selectin and neutrophil P-selectin glycoprotein ligand-1 (PSGL-1) [269]. Such interaction is enhanced following ADP-mediated platelet activation [263]. Platelet-neutrophil interactions are also enhanced in the presence of *P. gingivalis* in a preincubation time-dependent manner. Moreover, bacterial exposure to whole blood can trigger the neutrophil release of nuclear DNA, also known as neutrophil extracellular traps (NETs). Such release of NETs in response to *P. gingivalis* is known to be at least in part dependent on an interaction between activated platelets and neutrophils [263]. This implies that the interaction of *P. gingivalis* with the various blood cells does not only potentially alter their cell-specific functions in response to this pathogen but can also impact their physiologic cell-cell interactions.

Protease-activated receptors (PARs), members of the GPCR family, are characterized by a unique activation mechanism. The amino terminus of PARs is cleaved to expose an auto-activating tethered ligand that triggers intracellular signal transduction via an internal salt bridge formation [270]. Human platelets express two types of PARs, PAR-1, and PAR-4. These receptors are normally proteolytically activated by the serine protease thrombin as one of the mechanisms of platelet activation [271]. *P. gingivalis* expressed gingipains, however, can also cleave and activate PAR-1 and PAR-4 [40]. RgpA is up to six-fold more efficient in activating PAR-4 compared to thrombin [40]. Thrombin, however, is significantly more efficient at PAR-1 activation compared to either RgpA or RgpB [40]. The particular activation efficiency of PAR-1 by thrombin is likely due to a hirudin-like sequence contained within the exodomain of PAR-1, which binds with high affinity to the anion-binding exosite of thrombin [272]. Furthermore, cytosolic calcium levels are increased following the incubation of isolated platelets with purified RgpA or RgpB. Pretreatment of platelets with an anti–PAR-1 antibody abrogates this effect, supporting the role of arginine gingipain dependent PAR-1 cleavage in platelet calcium activities [40]. Similarly, the treatment of platelets with a protease inhibitor completely abolished this effect [40]. In this context, lower levels of RgpA are required to induce platelet aggregation compared to RgpB, emphasizing its higher efficiency at mediating platelet responses.

However, the proteolytic functions of gingipains are not solely responsible for mediating platelet aggregation. In the presence of *P. gingivalis*, platelet aggregation is observed in platelet-rich plasma (PRP) treated individually or in combination with inhibitors for Rgp or Kgp [273]. This suggests that other bacterial products may also mediate some platelet aggregating effects. Hgp44, an adhesin domain expressed at the C-termini of RgpA and Kgp, plays a role in hemagglutination and hemoglobin binding [27]. Incubating PRP with a mutant *P. gingivalis* strain deficient in adhesin domains only or with a strain deficient in Rgp, Kgp, and adhesin domains does not induce platelet aggregation [273]. However, platelet aggregating potential is restored when a recombinant Hgp44 is preincubated with either mutant strain prior to incubation with PRP. Furthermore, incubating PRP with *P. gingivalis* in the presence of anti-Fc_γ_RIIa mAb inhibits platelet aggregation [273]. Similarly, platelet aggregation can be somewhat reduced when PRP is incubated with *P. gingivalis* and an anti-glycoprotein (GP) Ibα mAb. However, aggregation of washed platelets, treated with gingipain deficient strains, is restored if anti-*P. gingivalis* immunoglobulin G (IgG) is added [273]. Taken together, *P. gingivalis* can induce platelet aggregation independent of gingipains via pathways that involve contributing roles from Fc_γ_RIIa, IgG, and GPIbα.

## 18. Limitations

This review represents the current understanding from a basic science perspective of the role of *P. gingivalis* in the pathogenesis of oral inflammatory processes. In addition, the potential impact on the functioning of certain blood cells, such as platelets and neutrophils, is also considered, particularly regarding their subsequent roles in increased prothrombotic risks. However, this review does not represent an encyclopedic compilation of everything currently known about this pathogen. Similarly, it does not detail the impact of this pathogen on every cell type that it may come in contact with and possibly infect. Of particular interest might be the effects of *P. gingivalis* on the cells and tissues of blood vessel walls. That would be a worthy topic for a review in its own right.

## 19. Conclusions

*P. gingivalis* is an opportunistic pathogen that infects the subgingival tissues of the oral cavity. Virulence factors, most notably arginine- and lysine-specific gingipains, play a central role in mediating *P. gingivalis* pathogenicity. Gingipains are involved with all aspects of *P. gingivalis*-induced infection, including nutrient acquisition essential for bacterial growth, tissue breakdown to facilitate bacterial invasion, and degradation of cytokines to disrupt the host inflammatory responses. Gingipains also contribute to *P. gingivalis*-mediated subversion of host immune responses by cleaving complement proteins and exploiting the TLR signaling pathway to attenuate neutrophil functions. Ineffective bacterial clearance reinforces persistent *P. gingivalis* infection and propagates chronic inflammation, driving the periodontitis pathophysiology. A growing body of evidence further suggests a potential circulatory prothrombotic role of *P. gingivalis*. Platelet activation and aggregation are observed in the presence of this periodontopathogen. Future studies should include further characterization of the thrombotic mechanisms triggered by *P. gingivalis* and its expressed virulence factors.

## Figures and Tables

**Table 1:** Virulence factors produced by *Porphyromonas gingivalis*.

Virulence Factor	Action	References
**Capsule**	Avoid detection by the immune system Contribute to *P. gingivalis* invasion of host tissue	[20,21]
**Fimbriae**	Contribute to *P. gingivalis* invasion of host tissue	[22]
Major fimbriae	Mediate *P. gingivalis* attachment to host tissue Induces remodeling of the actin cytoskeleton in gingival epithelial cells	[23,24]
Minor fimbriae	Triggers cytokine release by macrophages	[25,26]
**Gingipains**	Chemokine and cytokine degradation Nutrient (heme and peptide) acquisition Subversion of host immune response	[27,28,29,30]
RgpA	Contribute ECM attachment and fimbriae assembly Cleave ECM proteins and facilitate tissue invasion Degrade C3, C5a, and RBCs Bind to C4BP, resulting in decreased C3 and C4 protein function Cleave and activate FIX, FX, prothrombin, PAR-1, and PAR-4	[31,32,33,34,35,36,37,38,39,40]
RgpB	Mediate *P. gingivalis* binding to ECM proteins Involved with the assembly of fimbrial proteins Cleave and activate FIX, FX, PAR-1, and PAR-4	[40,41,42]
Kgp	Cleave ECM proteins and facilitate tissue invasion Bind to and degrade hemoglobin for heme acquisition Cleave C5a receptor	[31,43,44]

C3, complement component 3; C4, complement component 4; C4BP, complement component 4b binding protein; C5a, complement component 5a; ECM, extracellular matrix; FIX, factor IX; FX, factor X; PAR, protease-activated receptor; RBCs, red blood cells.

## Data Availability

This is a narative review that largely relies on published peer-reviewed research articles and discussions. Data is presented with citations of supporting source publications.

## Abbreviations

AAs: amino acids; BAR, SspB adherence region; C, component; C3bBb, C3 convertase complex; C4b2a, C3 convertase complex; C4BP, C4b-binding protein; C5aR, C5a receptor; CAMs, cell adhesion molecules; CCL, chemokine (C-C motif) ligand; CFU, colony-forming unit; CPS, capsular polysaccharides; CXCL, chemokine (C-X-C motif) ligand; DNA, deoxyribonucleic acid; ECM, extracellular matrix; ELAM-1, endothelial leukocyte adhesion molecule-1; Fap2, fibroblast activation protein 2; Fim, fimbrillin; FIX, factor IX; FIXa, activated factor IX; FVa, activated factor V; FVIIa, activated factor VII; FX, factor X; FXa, activated factor X; GP, glycoprotein; GCF, gingival crevicular fluid; GPCR, G protein-coupled receptor; HUVECs, human umbilical vein endothelial cells; ICAM-1 intercellular adhesion molecule-1; IgG immunoglobulin G; IL, interleukin; k_cat_/K_m_, catalytic efficiency; Kgp, lysine gingipain; LPS, lipopolysaccharide; mAb, monoclonal antibody; MALDI–TOF MS, matrix-assisted laser desorption ionization–time of flight mass spectrometry; MAPK, mitogen-activated protein kinase; MDCK, Madin-Darby canine kidney; Mfa, Minor fimbrial antigen; MMPs, matrix metalloproteinases; mRNA, messenger RNA; MYD88, Myeloid differentiation primary response 88; N-acetylglucosamine, 2-acetamido-2-deoxy-d-glucose; NFATc1, nuclear factor of activated T-cells, cytoplasmic 1; NGS, next generation sequence; OPG, osteoprotegerin; PAD, peripheral arterial disease; PARs, protease-activated receptors; PLC, phospholipase C; PMNs, polymorphonuclear leukocytes; PRP, platelet-rich-plasma; PSGL-1, P-selectin glycoprotein ligand-1; RadD, arginine (R)-inhibitable adhesin; RANK, receptor activator of nuclear factor κβ; RANKL, receptor activator of nuclear factor κβ ligand; RBCs, red blood cells; RgpA, arginine gingipain A; RgpB, arginine gingipain B; RNA, ribonucleic acid; ROS, reactive oxygen species; rRNA, ribosomal RNA; SRP, Scaling and root planing; Ssp, Streptococcal surface protein; TF, tissue factor; TLR, toll-like receptor; TNF, tumor necrosis factor; VCAM-1, vascular cell adhesion molecule-1; vWF, von Willebrand Factor.
